# Pathological complete response with nivolumab for recurrence of liver metastasis after gastrectomy of gastric cancer

**DOI:** 10.1186/s40792-023-01668-x

**Published:** 2023-05-19

**Authors:** Chen Jun, Suguru Yamauchi, Yukinori Yube, Hiroki Egawa, Yutaro Yoshimoto, Akira Kubota, Kenki Tsuda, Sanae Kaji, Hajime Orita, Shinichi Oka, Shinji Mine, Tetsu Fukunaga

**Affiliations:** 1 Department of Esophageal and Gastroenterological Surgery, Juntendo University Hospital, 3-1-3 Hongo, Bunkyo-Ku, Tokyo, 113-8431 Japan; 2 Department of Surgery, Koshigaya Municipal Hospital, 10-32 Higasikoshigaya, Koshigaya, Saitama 343-8577 Japan; 3 Department of Surgery, The Johns Hopkins University School of Medicine, Baltimore, MD 21287 USA

**Keywords:** Gastric cancer, Nivolumab, Complete response, Liver metastasis, PET–CT imaging

## Abstract

**Background:**

Advanced gastric cancer has an unfavorable prognosis and poor curability. Immune checkpoint inhibitors, such as nivolumab, have recently emerged as a potential solution for this aggressive disease. However, there is a lack of established evidence on the clinical efficacy of these agents, particularly in the perioperative period for advanced gastric cancer patients who are unresectable, recurrent, or preoperative. Despite the limited data available, there have been rare cases of dramatic therapeutic effects. In this study, we present a successful case of nivolumab treatment along with surgery.

**Case presentation:**

A 69-year-old female presented with pericardial discomfort and was diagnosed with advanced gastric cancer following upper gastrointestinal endoscopy. Laparoscopic distal gastrectomy with D2 lymph node dissection was performed, resulting in a final pathological diagnosis of Stage IIIA. The patient received postoperative adjuvant chemotherapy with oral S-1 therapy, but was found to have multiple liver metastases at 8 months postsurgery. Weekly paclitaxel and ramucirumab therapy was initiated, but the patient experienced adverse side effects, leading to the discontinuation of treatment. Nivolumab monotherapy was then administered for 18 cycles, resulting in a partial therapeutic response and PET–CT revealed a complete metabolic response. However, the patient developed a Grade 3 pemphigoid as an immune-related adverse event, leading to the cessation of nivolumab. The patient underwent laparoscopic partial hepatectomy. Postoperative pathology showed no residual tumor cells, indicating a complete response. At present, 25 months after surgery, the patient was alive without recurrence.

**Conclusion:**

In this report, we present a case of gastric cancer with liver metastatic recurrence, in which a complete pathological response was achieved with nivolumab treatment. Although determining whether surgical intervention is necessary following successful drug treatment can be challenging, PET–CT imaging may be useful in decision-making regarding surgical treatment.

## Background

The prognosis for unresectable advanced or recurrent gastric cancer is grim, and current treatment options are limited [1, 2]. The standard treatment for Stage IV gastric cancer is chemotherapy, and there is currently no high-quality evidence on the effectiveness of surgical intervention. However, recent years have seen the emergence of new anticancer agents, including molecular-targeted drugs and immune checkpoint inhibitors. Although the response to anticancer drugs in advanced gastric cancer patients is typically poor, some rare cases have shown a dramatic therapeutic response. In ATTRACTION-2, nivolumab treatment resulted in a partial response (PR) in 10.8% of patients and a complete response (CR) in 1.1% of patients, underscoring the need to consider surgical intervention to further improve prognosis [3]. However, numerous issues remain unresolved, including treatment indications, timing, surgical techniques, and postoperative treatment. We present a case of a patient with recurrent liver metastasis after gastrectomy, who achieved a pathological CR through laparoscopic partial hepatectomy after treatment with nivolumab. This patient has survived for a prolonged period, highlighting the potential efficacy of nivolumab treatment in cases of advanced gastric cancer.

## Case presentation

**Fig. 1:**
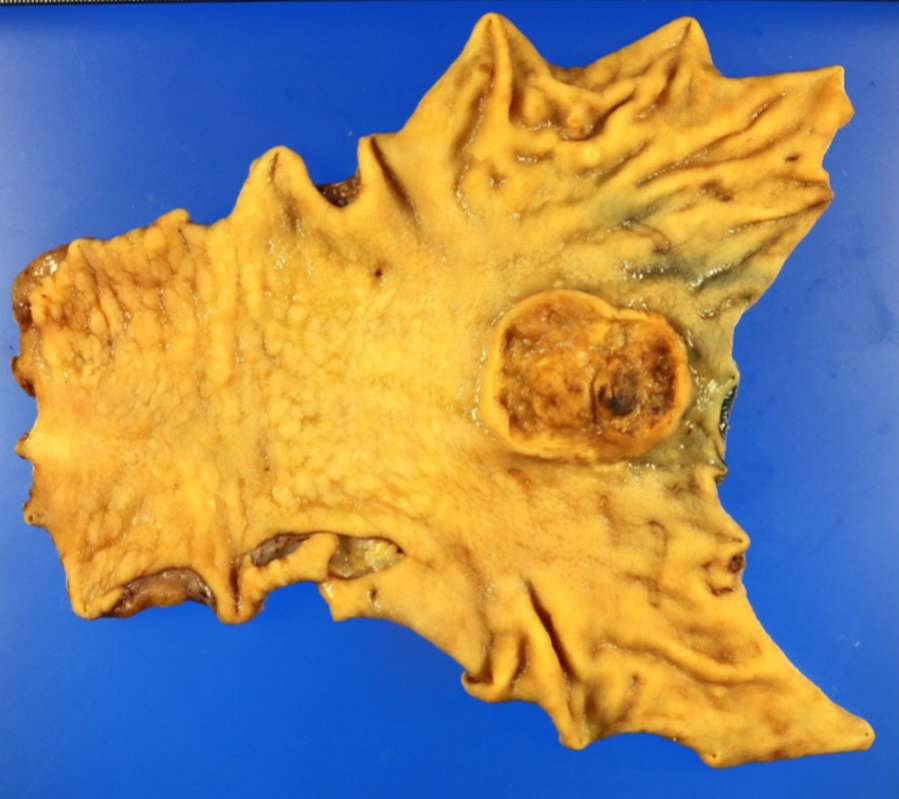
Resected gastric specimen. A 46 × 38 mm Borrmann type 2 gastric cancer was treated by laparoscopic distal gastrectomy with no residual tumor

**Fig. 2:**
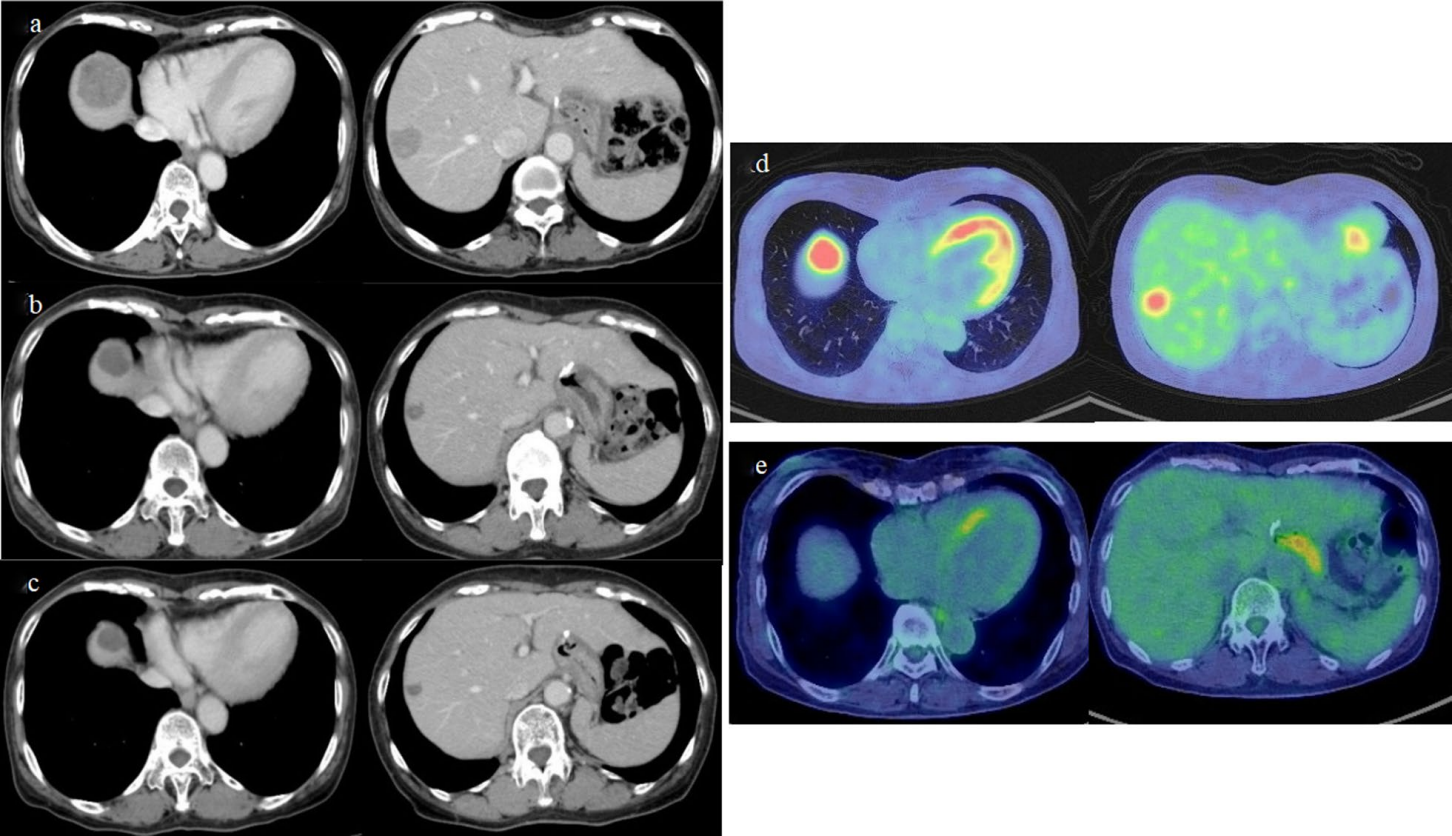
Contrast-enhanced CT and PET–CT during nivolumab treatment. **a** CT before nivolumab treatment. Liver metastases were 41 × 39 mm in segment 4 and 24 × 22 mm segment 8. **b** CT after 8 courses of nivolumab. The target lesion of liver metastasis achieved a therapeutic partial response. **c** CT after 18 courses of nivolumab. Liver metastases had shrunk to 24 × 22 mm in segment 4 and 11 × 9 mm in segment 8. **d** PET–CT at the time of recurrence and before nivolumab administration. No evidence of intrahepatic metastases other than two recurrent hepatic metastases was noted on CT. **e** PET–CT after 18 courses of nivolumab. No abnormal fluorodeoxyglucose (FDG) accumulation in liver metastases was shown, and no new metastases appeared

A 69-year-old woman presented with pericardial discomfort and was subsequently diagnosed with advanced gastric cancer after upper gastrointestinal endoscopy. The patient underwent laparoscopic distal gastrectomy with D2 lymph node dissection, and the final pathological diagnosis was U, Less, Type 2, 46 × 38 mm, adenocarcinoma with enteroblastic differentiation, pT4a, INFa, Ly0, V0, pPM0, pDM0, pN2, and pStageIIIA (Japanese Classification of Gastric Carcinoma[4]) (Fig. 1). Human epidermal growth factor type 2 was negative, combined positive score of programmed cell death ligand 1 was 5 or higher and microsatellite instability was negative. The patient received oral S-1 (80 mg/m^2^ twice a day for 1–28 days) as postoperative adjuvant chemotherapy, but liver metastases were observed 8 months later in segments 4 (41 × 39 mm) and 8 (24 × 22 mm). The location and number of metastases were confirmed by contrast-enhanced computed tomography (CT), magnetic resonance imaging (MRI) and positron emission tomography (PET)–CT. There was no elevation observed in the tumor markers carcinoembryonic antigen (CEA) and carbohydrate antigen 19-9 (CA19-9). The patient was switched to weekly paclitaxel plus ramucirumab, but Grade 4 neutropenia was observed during the first cycle, and nivolumab monotherapy was initiated (3 mg/kg intravenously every 2 weeks) after consultation with the patient (Fig. 2).

After eight cycles, the target lesion of liver metastasis achieved a PR by Response Evaluation Criteria in Solid Tumors (RECIST), and nivolumab treatment was continued. After 18 cycles, liver metastases had shrunk to 24 × 22 mm in segment 4 and 11 × 9 mm in segment 8, and PET–CT showed no abnormal fluorodeoxyglucose (FDG) accumulation in liver metastases, and no new metastases appeared. CEA and CA19-9 did not show any significant changes within the normal range during the course of treatment. However, at this point, the patient developed a Grade 3 immune-related adverse event (irAE), a pemphigoid skin disorder, and the nivolumab treatment was discontinued.

**Fig. 3:**
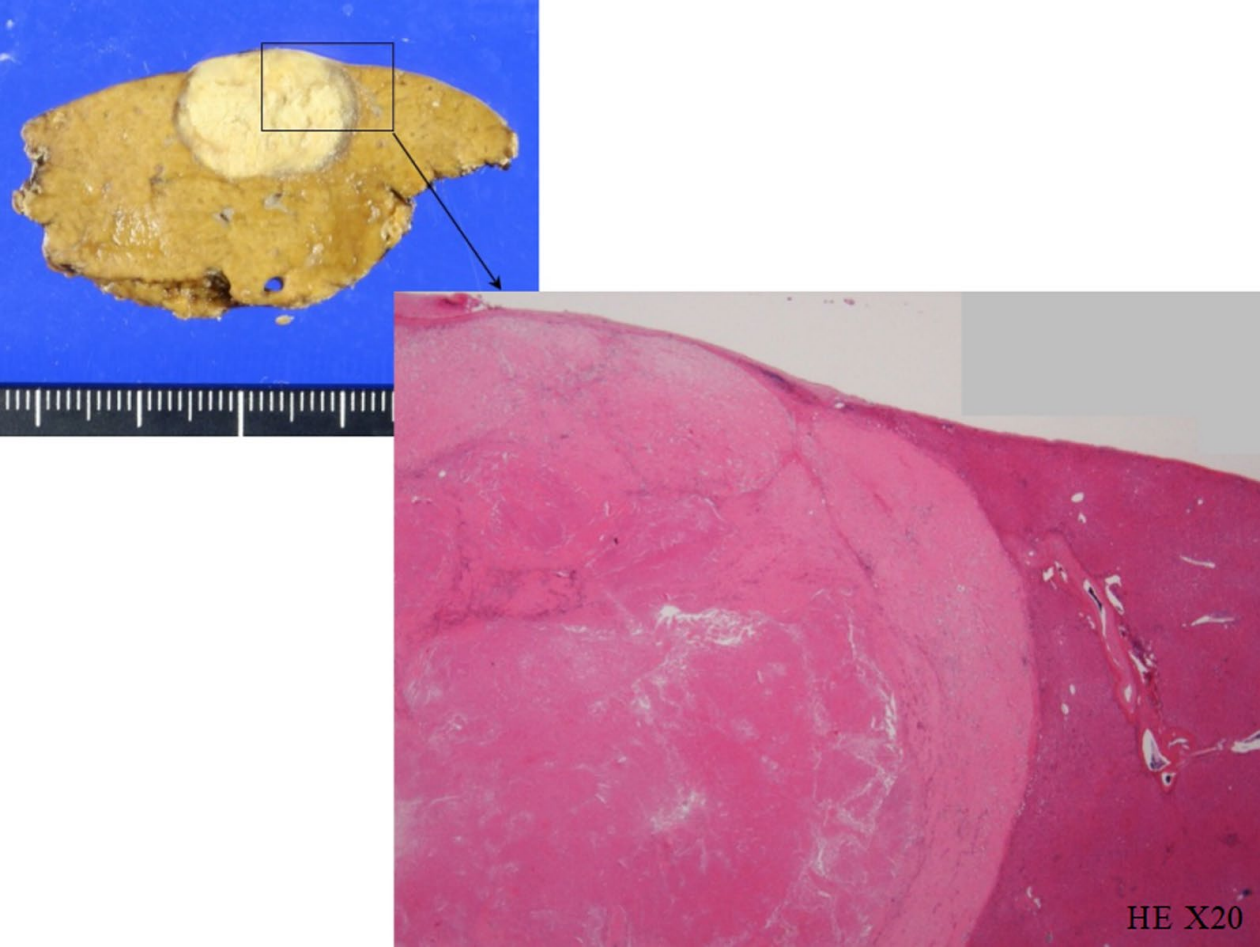
Pathological findings of resected liver metastases. The pathological findings showed that there was only mucus accumulation from the submucosa to the serosa with no residual tumor cells that could be considered viable, determined to be pathological complete response

As the metastases were controllable with anticancer agents, no new lesions appeared, and the patient’s performance status was maintained, it was decided to resect the liver metastases. The patient underwent laparoscopic partial hepatectomy for segments 4 and 8, resulting in pathological CR (Fig. 3). After a medication withdrawal period due to the surgery, the skin disorder had resolved, and postoperative chemotherapy with nivolumab was resumed. However, due to a recurrence of the skin disorder, the chemotherapy was immediately stopped. The patient was followed up without treatment, and no recurrence findings were observed for 25 months postoperatively.

## Discussion

This is the first case of long-term survival with pathological CR after nivolumab therapy and resection for liver metastases after gastrectomy for gastric cancer. While liver metastasis is often thought to be usually a predictor of a poor response to nivolumab, strategic planning without compromising treatment time may lead to a better patient outcome [5]. Based on prior clinical trial data, the median survival period for unresectable advanced or recurrent gastric cancer is roughly 6–13 months, and the prognosis is exceedingly poor, with few therapeutic choices. Systemic chemotherapy is indicated for Stage IV gastric cancer with liver metastases because surgical excision of liver metastases of gastric cancer is still controversial, and in many cases, multiple recurrences of the liver metastases may occur after hepatectomy [6, 7]. The Japanese Guidelines for the Treatment of Gastric Cancer also do not clearly define the surgical treatment of liver metastases, only suggesting that surgical resection is weakly recommended when the number of metastases is small and there are no other noncurative factors. Yoshida et al. proposed a biological classification for the optimal therapeutic strategy for patients with Stage IV gastric cancer and indicated conversion therapy [8]. This treatment plan is roughly split into two groups, based on whether there is large-scale peritoneal spread, and four groups, based on whether metastases can be removed. In the classification of liver metastases without gross peritoneal dissemination, one metastasis is designated Category 1, and more than one metastasis or lesions of > 5 cm or close to a vein or portal vein are designated Category 2. Category 1 is resection after neoadjuvant chemotherapy, and Category 2 is considering surgery if resection is not possible after PR and CR results with intensive chemotherapy.

Eight months following surgery, the patient was classified as Category 2 because there were at least two recurring liver metastases visible on contrast-enhanced CT, MRI and PET–CT. Intensive chemotherapy with S-1, paclitaxel, and ramucirumab was tried but failed; thus, the patient was given nivolumab. The efficacy of nivolumab was not satisfactory in the ATTRACTION-2 trial, with a median overall survival of 5.3 months, median progression-free survival of 1.6 months, and a response rate of 11.2%, although in the 2-year follow-up report, the survival of responders who achieved CR or PR median was 26.6 months, and the 3-year OS rate was 35.5%, and it is necessary to consider how to incorporate surgical treatment in these patients to improve their prognosis[3, 9]. This patient was treated during the period when the Japanese Guidelines for the Management of Gastric Cancer 2018 suggested nivolumab as third-line chemotherapy for patients with unresectable advanced or recurrent gastric cancer [10].

Here, the most difficult strategic decision for the patient was whether to undergo surgery. Although known metastases could be controlled with nivolumab and CT, and PET–CT results showed no new intrahepatic or distant metastases, the appearance of irAE due to skin lesions made it impossible to continue chemotherapy. Due to the need for a drug interruption period, the patient decided to undergo surgery. Consequently, pathological CR was confirmed by resection of the liver metastatic lesions. However, even if the patient had not undergone surgery and had been followed up, a similar good prognosis could have been achieved in terms of the clinical course. With the backward-looking clinical examination factors that may have prevented surgery, PET–CT results could support a treatment strategy in the future when similar cases are experienced. A complete metabolic response (CMR) is defined as the full elimination of FDG accumulation in PET–CT, which is indistinguishable from buildup in surrounding normal tissue [11]. This is equivalent to a CR in RECIST and is widely used for functional metabolic and anatomic/morphologic imaging of various types of malignant tumors and metastatic lesions. It can also provide several metabolic parameters to distinguish metabolically active from inactive tumor tissue and has been reported to be useful as a therapeutic response determination in some tumors, although rarely in the gastric cancer area [12]. However, note that CMR on PET–CT in gastric cancer at this time cannot be interpreted as a completely equivalent finding to pathologically confirmed CR. The clinical course of this case may support the possibility that if PET–CT shows a response comparable to CMR in a case for which chemotherapy cannot be continued and aggressive surgery should not be considered, then strict follow-up might be an option.

With the new clinical development of immune checkpoint inhibitors, there have been some reports of nivolumab being used successfully in the perioperative period. We used PubMed to search for case reports in English with the key terms “Gastric Cancer”, “Stage IV”, “Nivolumab”, and “CR”, and summarized them in Table 1 [9, 13–24]. The definition of CR in this search also included clinical and pathological CR. Thirteen instances have been documented to date, with the frequency of complaints rising in recent years. The majority of the patients were treated with nivolumab for Stage IV gastric cancer with distant metastasis at the time of initial diagnosis. The only case in which nivolumab was indicated for recurrent liver metastasis after gastrectomy, as in this case, was reported by Namikawa et al. [24] Thus far, this is the first case in which CR was acquired pathologically from a metastatic lesion. The number of courses of nivolumab administered before surgery ranges from 5 to 48, indicating that the best physical and oncological conditions for transition to surgery are known and practiced on a patient-by-patient basis. About half of the patients continue to receive nivolumab as adjuvant therapy after surgery, but further discussion is needed on the merits and efficacy of nivolumab as a postoperative treatment, as well as the necessary duration of administration. Such instances are predicted to grow in the future as more cases of nivolumab treatment for unresectable advanced gastric cancer in the early phases of treatment are documented, as well as its availability for use in neoadjuvant chemotherapy. Therefore, it is essential that more case series and prospective studies are published to help improve patient survival outcomes.

**Table 1:** Summary of complete response cases with nivolumab treatment for Stage IV gastric cancer

References	Year	Age	Sex	Initial treatment	Metastasis (recurrence)	Number of chemotherapy regimens prior to nivolumab	Course of nivolumab	Post-nivolumab surgery	Post nivolumab chemotherapy	Adverse events	Prognosis
Dai et al. [13]	2022	66	M	Palliative surgery	Liver	1	8	Hepatectomy	None	Immune pneumonia	32 months
Toyota et al. [14]	2022	73	M	Chemotherapy	Liver (invasion)	None	5	Distal gastrectomy and hepatectomy	S-1	None	N/A
Pan et al. [15]	2022	45	M	Chemotherapy	Liver, lung, lymph nodes	3	5	Total gastrectomy	Nivolumab	Exfoliative dermatitis eruption	4 years
Takami et al. [16]	2021	70	F	Total gastrectomy	Peritoneal dissemination, lymph nodes	2	54	None	Nivolumab	None	7 years
Watanabe et al. [17]	2021	73	M	Chemotherapy	Liver	2	31	Total gastrectomy	None	None	43 months
Kumamoto et al. [18]	2021	69	M	Chemotherapy	Lymph nodes	2	8	Esophagectomy	None	None	Over 12 months
Komo et al. [19]	2021	69	F	Chemotherapy	Peritoneal dissemination	2	9	None	None	Immune pneumonia	Over 20 months
Matsumoto et al. [9]	2020	63	F	Chemotherapy	Liver, lung	2	26	Distal gastrectomy	Nivolumab	None	41 months
Kuhara et al. [20]	2020	60	F	Chemotherapy	Lymph nodes	2	39	None	Nivolumab	Rash	Over 48 months
Toyota et al. [21]	2020	75	M	Chemotherapy	Peritoneal dissemination	2	23	Distal gastrectomy	None	None	Over 20 months
Toyota et al. [22]	2020	70	M	Chemotherapy	Pancreas and spleen (invasion), lymph nodes	2	24	Total gastrectomy with distal pancreatosplenectomy	None	Rash, gland hypofunction	Over 20 months
Kashima et al. [23]	2019	25	M	Chemotherapy	Lymph nodes	1	48	Total gastrectomy	Nivolumab	None	Over 60 months
Namikawa et al. [24]	2018	77	M	Total gastrectomy	Spleen, liver	2	16	None	Nivolumab	None	28 months

N/A: not available

## Conclusion

Here, we report a case of recurrent gastric cancer with liver metastasis, where complete pathological response was achieved with nivolumab. PET–CT could aid in making treatment strategy decisions about the need for surgical intervention.

## Data Availability

Data sharing is not applicable to this article as no datasets were generated or analyzed during the current study.

## Abbreviations

PR: Partial response
CR: Complete response
CT: Computed tomography
MRI: Magnetic resonance imaging
PET: Positron emission tomography
CEA: Carcinoembryonic antigen
CA19-9: Carbohydrate antigen 19–9
RECIST: Response Evaluation Criteria in Solid Tumors
FDG: Fluorodeoxyglucose
irAE: Immune-related adverse event
CMR: Complete metabolic response

## References

[1] Ito S, Oki E, Nakashima Y, Ando K, Hiyoshi Y, Ohgaki K, Saeki H, Morita M, Sakaguchi Y, Maehara Y (2015). Clinical significance of adjuvant surgery following chemotherapy for patients with initially unresectable stage IV gastric cancer. Anticancer Res.

[2] Kanda T, Yajima K, Kosugi S, Ishikawa T, Ajioka Y, Hatakeyama K (2012). Gastrectomy as a secondary surgery for stage IV gastric cancer patients who underwent S-1-based chemotherapy: a multi-institute retrospective study. Gastric Cancer.

[3] Chen LT, Satoh T, Ryu MH, Chao Y, Kato K, Chung HC, Chen JS, Muro K, Kang WK, Yeh KH, Yoshikawa T, Oh SC, Bai LY, Tamura T, Lee KW, Hamamoto Y, Kim JG, Chin K, Oh DY, Minashi K, Cho JY, Tsuda M, Sameshima H, Kang YK, Boku N (2020). A phase 3 study of nivolumab in previously treated advanced gastric or gastroesophageal junction cancer (ATTRACTION-2): 2-year update data. Gastric Cancer.

[4] JGC Association (2011). Japanese classification of gastric carcinoma: 3rd english edition. Gastric Cancer..

[5] Bearz A, Perin T, Cancian L, Berto E, Sartor I, Tirelli U (2016). Immune checkpoint inhibitors and response analysis: a tough challenge. A case report. BMC Res Notes.

[6] Kinoshita T, Kinoshita T, Saiura A, Esaki M, Sakamoto H, Yamanaka T (2015). Multicentre analysis of long-term outcome after surgical resection for gastric cancer liver metastases. Br J Surg.

[7] Oki E, Tokunaga S, Emi Y, Kusumoto T, Yamamoto M, Fukuzawa K, Takahashi I, Ishigami S, Tsuji A, Higashi H, Nakamura T, Saeki H, Shirabe K, Kakeji Y, Sakai K, Baba H, Nishimaki T, Natsugoe S, Maehara Y (2016). Surgical treatment of liver metastasis of gastric cancer: a retrospective multicenter cohort study (KSCC1302). Gastric Cancer.

[8] Yoshida K, Yamaguchi K, Okumura N, Tanahashi T, Kodera Y (2016). Is conversion therapy possible in stage IV gastric cancer: the proposal of new biological categories of classification. Gastric Cancer.

[9] Matsumoto R, Arigami T, Matsushita D, Okubo K, Tanaka T, Yanagita S, Sasaki K, Noda M, Kita Y, Mori S, Kurahara H, Ohtsuka T (2020). Conversion surgery for stage IV gastric cancer with a complete pathological response to nivolumab: a case report. World J Surg Oncol.

[10] Japanese gastric cancer treatment guidelines (2018). Japanese gastric cancer treatment guidelines 2018 (5th edition). Gastric Cancer.

[11] Hirata K, Tamaki N (2021). Quantitative FDG PET assessment for oncology therapy. Cancers (Basel).

[12] Vitolo U, Trněný M, Belada D, Burke JM, Carella AM, Chua N, Abrisqueta P, Demeter J, Flinn I, Hong X, Kim WS, Pinto A, Shi YK, Tatsumi Y, Oestergaard MZ, Wenger M, Fingerle-Rowson G, Catalani O, Nielsen T, Martelli M, Sehn LH (2017). Obinutuzumab or rituximab plus cyclophosphamide, doxorubicin, vincristine, and prednisone in previously untreated diffuse large B-cell lymphoma. J Clin Oncol.

[13] Dai P, Rao X, Zhang X, Qiu E, Wu G, Lin Y, Li S, Li Z, Cai Z, Han S (2022). Case report: complete remission of a patient with metastatic gastric cancer treated with nivolumab combined with chemotherapy after palliative surgery. Front Immunol.

[14] Toyota K, Hashimoto Y, Sakashita Y, Yokoyama Y, Murakami Y, Takahashi S, Miyamoto K (2022). Pathological complete response to Nivolumab, S1, Oxaliplatin, and radiation in a patient with gastric cancer: a case report. J Gastrointest Cancer.

[15] Pan Y, Lu L, Liu H, Chen D, Han N, Yao R, Wang X, Gao X, Yu J, Chen L, Zhou F, Hao G, Lu Y, Li M, He G, Kang F, Li Z, Tang Y, Zhang J, Wei L, Nie Y (2022). Case report: Long response to PD-1 blockade after failure of trastuzumab plus chemotherapy in advanced Epstein-Barr virus-associated gastric cancer. Front Immunol.

[16] Takami T, Yasuda K, Uozumi N, Musiake Y, Shintani H, Kataoka N, Yamaguchi T, Makimoto S (2021). Confirmed complete response to nivolumab for advanced gastric cancer with peritoneal dissemination: a case report. J Med Case Rep.

[17] Watanabe H, Fujikawa H, Komori K, Kano K, Takahashi K, Yamada T, Inokuchi Y, Machida N, Yokose T, Rino Y, Masuda M, Ogata T, Oshima T (2021). Successful conversion surgery for stage IV gastric cancer after nivolumab monotherapy as third-line chemotherapy. Case Rep Gastroenterol.

[18] Kumamoto T, Tomita T, Hojo Y, Nakamura T, Kurahashi Y, Ishida Y, Miwa H, Hirota S, Shinohara H (2021). Pathological complete response and successful conversion surgery after nivolumab therapy for stage IV oesophagogastric junction cancer. In Vivo.

[19] Komo T, Suzuki T, Tazawa H, Sada H, Morimoto H, Shimada N, Hadano N, Onoe T, Sudo T, Shimizu Y, Tashiro H (2021). Clinical complete response after nivolumab administered as a third-line treatment for unresectable advanced gastric cancer with peritoneal dissemination: a case report. Int J Surg Case Rep.

[20] Kuhara Y, Ninomiya M, Hirahara S, Doi H, Kenji S, Toyota K, Yano R, Kobayashi H, Hashimoto Y, Yokoyama Y, Sakashita Y, Miyamoto K (2020). A long-term survival case of unresectable gastric cancer with multidisciplinary therapy including immunotherapy and abscopal effect. Int Cancer Conf J..

[21] Toyota S, Orita H, Fukuyama Y, Motoyoshi S, Kawanami S, Maeda S, Kuramitsu E, Ichimanda M, Nagamatsu S, Nagata S, Kai S, Korenaga D, Mori M (2020). Successful conversion surgery following chylous ascites after nivolumab for advanced gastric cancer. In Vivo.

[22] Toyota S, Naito H, Motoyoshi S, Nakanishi R, Oki E, Orita H, Korenaga D (2020). Extended total gastrectomy after nivolumab for unresectable multivisceral invasive gastric cancer. Surg Case Rep.

[23] Kashima S, Tanabe H, Tanino M, Kobayashi Y, Murakami Y, Iwama T, Sasaki T, Kunogi T, Takahashi K, Ando K, Ueno N, Moriichi K, Fukudo M, Tasaki Y, Hosokawa M, Mizukami Y, Fujiya M, Okumura T (2019). Lymph node metastasis from gastroesophageal cancer successfully treated by nivolumab: a case report of a young patient. Front Oncol.

[24] Namikawa T, Ishida N, Tsuda S, Fujisawa K, Munekage E, Iwabu J, Munekage M, Uemura S, Tsujii S, Maeda H, Kitagawa H, Kobayashi M, Hanazaki K (2018). Successful treatment of liver metastases arising from early gastric cancer achieved clinical complete response by nivolumab. Surg Case Rep.

